# Surgery with leukocyte and platelet-rich fibrin (L-PRF) vs. surgery alone for medication-related osteonecrosis of the jaw: A randomized controlled trial

**DOI:** 10.4317/jced.62824

**Published:** 2025-07-01

**Authors:** Emilio Andres Ramos, Juan Manuel Muiño, Maximiliano Diamante, Alejo Echagüe, Sebastian Ariel Puia, Diego Caruso

**Affiliations:** 1 Department of Oral and Maxillofacial Surgery I, Facultad de Odontología, Universidad de Buenos Aires, Ciudad Autónoma de Buenos Aires, Argentina; 2 Department of Oral and Maxillofacial Surgery, Hospital Dr. César Milstein, Ciudad Autónoma de Buenos Aires, Argentina; 3 Instituto de Investigaciones Médicas Dr Alfredo Lanari, Facultad de Medicina, Universidad de Buenos Aires, Ciudad Autónoma de Buenos Aires, Argentina; 4 Department of Internal Medicine, Hospital Dr. César Milstein, Ciudad Autónoma de Buenos Aires, Argentina

## Abstract

**Background:**

Multiple studies have reported high resolution rates of medication-related osteonecrosis of the jaw (MRONJ) with surgical treatment combined with platelet concentrates. However, no randomized controlled trials have been published to date comparing surgery combined with L-PRF to surgery alone.

**Material and Methods:**

We conducted a multicenter, open-label, parallel-group, randomized controlled trial to assess the effect of leukocyte- and platelet-rich fibrin (L-PRF) as an adjunct to surgery for the treatment MRONJ. Patients were randomly assigned to either surgery combined with L-PRF or surgery alone. The primary outcome was the proportion of patients achieving complete resolution of MRONJ lesions six months postoperatively. Complete resolution rates were compared using the chi-square (X²) test, and relative risk (RR) with 95% confidence intervals (CI) was calculated. Secondarily, we evaluated baseline quality of life using the EQ-5D-3L questionnaire and assessed the impact of L-PRF on quality-of-life measures.

**Results:**

A total of 41 participants were included in the study. Nineteen participants underwent surgery with L-PRF, while 22 underwent surgery alone. At the six-month follow-up, complete resolution was observed in 92% (11/12) of patients in the experimental group and 83% (15/18) in the control group (RR 1.1, 95% CI 0.84–1.43, *p*=0.51). The most affected domains in the baseline quality-of-life assessment were anxiety/depression and pain/discomfort. No statistically significant differences were observed between groups in any quality-of-life domain at six months.

**Conclusions:**

Our findings suggest that the use of L-PRF as an adjunct to surgery does not provide a significant clinical benefit compared to surgery alone.

** Key words:**Platelet-rich fibrin, quality of life, osteonecrosis, randomized controlled trial.

## Introduction

Medication-related osteonecrosis of the jaws (MRONJ) is a drug-induced adverse reaction characterized by the progressive destruction of bone tissue in the maxillofacial region due to exposure to antiresorptive and/or antiangiogenic medications (1,2). Since its first description in the medical literature in 2003, various treatment alternatives have been proposed (3,4). However, there are no universally accepted strategies for its management (5,6). Leukocyte- and platelet-rich fibrin (L-PRF) is an autologous platelet concentrate composed of platelets, leukocytes, and stem cells embedded in a three-dimensional fibrin matrix, capable of releasing a wide array of growth factors that promote wound healing and tissue repair processes (7). Multiple studies have been published showing high rates of resolution of MRONJ cases with surgical treatment combined with L-PRF (8). However, no randomized controlled trials have been published to date comparing surgery combined with L-PRF to surgery alone. The aim of this study was to compare surgery combined with L-PRF to surgery alone in the treatment of patients with MRONJ.

## Material and Methods

We conducted a multicenter, open-label, parallel-group, randomized controlled trial at Dr. César Milstein Hospital, Argentina, and at the School of Dentistry, University of Buenos Aires, Argentina. The study was approved by the Research Ethics Committee of Dr. César Milstein Hospital (File number: 2018-01) and was conducted according to the ethical principles for medical research in human participants, as outlined in the 2013 Declaration of Helsinki. All participants provided written informed consent in accordance with established guidelines.

We included patients over 18 years of age diagnosed with MRONJ according to the criteria established by the American Association of Oral and Maxillofacial Surgeons (1) . We excluded patients who refused to participate, those who were not candidates for surgery due to their general condition, those who had received radiation therapy in the head and neck area, and those with neoplastic involvement of the oral cavity or in terminal stages of cancer with a life expectancy of less than six months.

Participants were randomized 1:1 in blocks of two, four, and six to surgery with L-PRF or surgery alone. The groups were stratified by MRONJ stage. The randomization list was generated using the platform www.sealedenvelope.com. The randomization sequence was concealed in numbered, opaque, and sealed envelopes, which were handled by personnel not involved in any other stage of the study. Both participants and the surgeon were not masked to the treatment group. The study was masked for outcome assessment. That is, the investigators responsible for the postoperative clinical evaluations were unaware of which treatment group each participant belonged to.

Both groups underwent conservative treatment prior to surgery, including mouth rinses with 0.12% chlorhexidine digluconate, analgesics, and antibiotic therapy in cases of infection. The antibiotic regimen consisted of amoxicillin 500 mg every 8 hours, amoxicillin-clavulanic acid 1 g every 12 hours, or, for penicillin-allergic patients, clindamycin 300 mg every 6 hours, all administered orally. Regardless of the presence of infection, all patients received antibiotic therapy at least one week prior to surgery. The discontinuation of the antiresorptive medication was determined individually for each patient.

The surgical procedure was performed under local anesthesia, intravenous sedation or general anesthesia, depending on the extent of the lesion and the patient’s general condition. Our surgical protocol included exposure of the bone lesion and ostectomy using manual and rotary instruments under constant irrigation with gentamicin-saline solution. The extent of the ostectomy was carried out until bleeding from the bone margins was evident. The teeth within the area of osteonecrosis were extracted. In all cases, bone tissue samples were collected for histopathological analysis, along with specimens for microbiological study. In cases of chronic sinusitis associated with the MRONJ lesion, a functional endoscopic maxillary sinus surgery was carried out during the same procedure. In patients with mandibular fractures or those at risk of pathological mandibular fracture, rigid fixation with osteosynthesis plates was performed via a transoral, cervical, or combined approach.

For L-PRF preparation, approximately 10 to 20 ml of blood was collected through a needle puncture using a vacuum collection system from a vein in the arm fold or another available vein. After collection, centrifugation was performed at 1200 rpm for 10 minutes at room temperature. After confirming the product visually, the tube was opened and presented to the surgeon, who, using sterile forceps, removed and separated the PRF from the rest of the clot to place it at the surgical site. Immediately after, we proceeded with tension-free closure of the mucoperiosteal flaps using 3.0 absorbable sutures. The sutures were removed after 10 days. Patients who continued to show bone exposure, infection, pain or cutaneous fistula after surgery received conservative treatment. Only those cases with severe overall health compromise or evident impairment of the patient’s quality of life underwent further surgical procedures. Follow-up visits were scheduled one and two weeks after surgery, and then once a month for six months. We carried out long-term follow-up evaluations, defined as those occurring after six months.

The primary outcome was the proportion of patients achieving complete resolution of MRONJ lesions at six months postoperatively, defined as the absence of pain (Numeric Pain Rating Scale= 0), absence of infection, absence of bone exposure (bone exposure= 0 mm), and absence of extraoral fistula. The presence of one or more of these clinical findings was categorized as “absence of complete resolution”. Each parameter (pain, infection, bone exposure, and extraoral fistula) was individually recorded to determine the effect of the intervention on each of them. Pain was evaluated using the Numeric Pain Rating Scale (NPRS) ranging from 0 to 100, where 0 represented “no pain” and 100 “the worst possible pain.” Infection was defined as the presence of purulent discharge and/or fever, swelling, pain, warmth, and redness on physical examination. Bone exposure was measured using a flexible millimeter-scale ruler. An extraoral fistula was defined as the presence of an extraoral path of infection that communicates the oral cavity and the skin. For the evaluation of the primary outcome, we considered patients as the unit of analysis. In patients with more than one lesion, we selected the most advanced-stage lesion for analysis of the primary outcome. We also evaluated the proportion of MRONJ lesions achieving complete resolution in each group at six months, using the same previously defined criteria.

The secondary outcomes were the proportion of patients achieving complete resolution in each group at the last follow up visit (defined as the last clinical follow-up the patient attended), the baseline quality of life of included patients using the EQ-5D-3L questionnaire (9) and the effect of the intervention on quality of life six months postoperatively.

Adverse events were continuously monitored from the time of consent until the six-month follow-up visit. Serious adverse events were defined as those requiring hospitalization, life-threatening events, those resulting in partial or total disability, or those leading to patient death.

The sample size calculation was based on an estimated difference for each treatment group: 75% for surgery alone and 95% for surgery combined with L-PRF (10,11). Considering an alpha error of 0.05 and a beta error of 0.2, the required sample size was 51 patients per group. To account for an anticipated 10% loss to follow-up, the final estimated sample size was 115 patients.

Proportions were compared using the Chi-square test or Fisher’s exact test, depending on data distribution, and the relative risk (RR) with 95% confidence intervals (95% CI) was calculated. Continuous variables were analyzed using Student’s t-test or the Wilcoxon test, depending on data distribution. A *p-value* <0.05 was considered statistically significant.

All statistical analyses were performed using STATA 18 (STATA Corp, TX, USA). Adverse events were reported descriptively.

## Results

**Figure 1:**
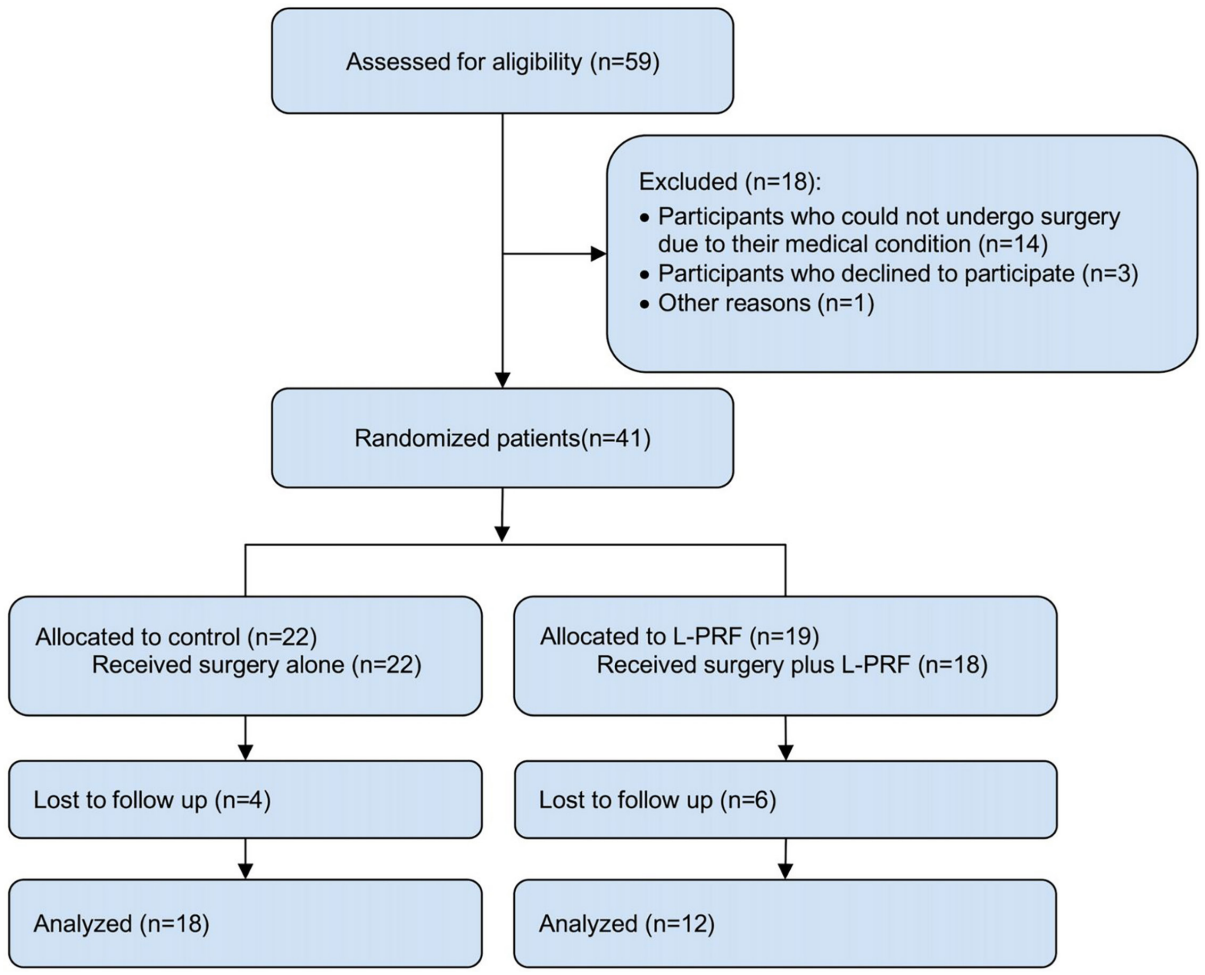
Flow of participants through each stage of the study.

A total of 59 patients diagnosed with MRONJ were evaluated between June 2018 and December 2023. Patient recruitment was terminated before reaching the planned sample size due to feasibility limitations. Given the observed recruitment rate and available resources, it was determined that achieving the target enrollment was not feasible within the study’s operational constraints. Figure 1 shows the flow of participants through each stage of the trial.

Baseline characteristics of included participants and baseline quality of life are presented in  Table 1.

**Figure 2:**
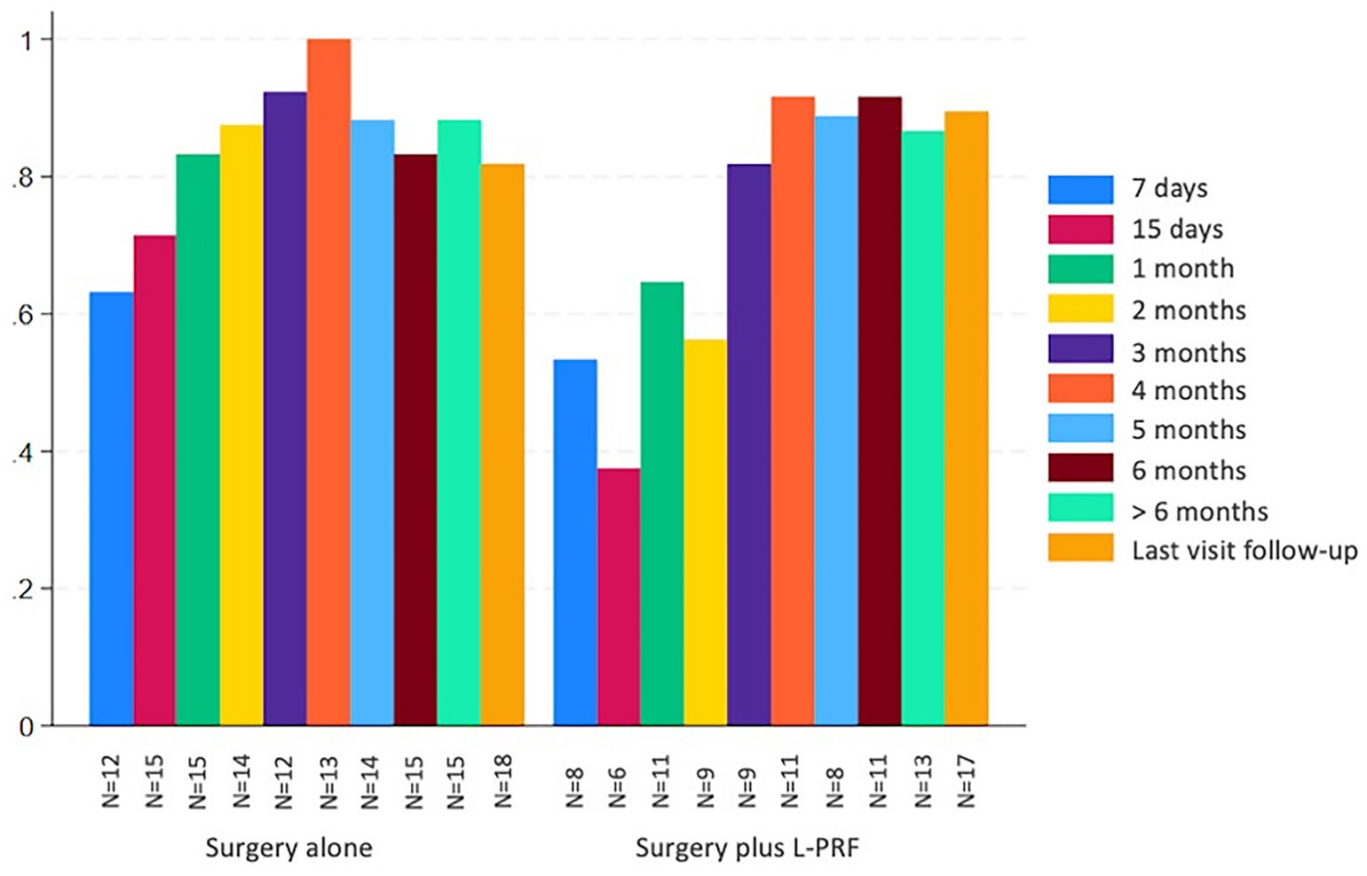
Complete resolution at different follow-up time points.

The proportion of patients achieving complete resolution at six months of follow-up was 92% (11/12) in the experimental group and 83% (15/18) in the control group (RR 1.1, 95% CI 0.84– 1.43, *p*=0.51) ( Table 2). Figure 2 illustrates the proportion of patients with complete resolution in each group at different follow-up time points.

No statistically significant differences were observed between the two groups in any of the individual clinical domains at six months, including pain, bone exposure, infection, and cutaneous fistula ( Table 3).

At the last follow-up visit, the proportion of patients achieving complete resolution was 92% (17/19) in the experimental group and 82% (18/22) in the control group (RR 1.094, 95% CI 0.85–1.04, *p*=0.49).

No statistically significant differences were detected between the experimental and control groups at six months across any domains of the EQ-5D-3L quality of life questionnaire ( Table 4).

Twenty one adverse events occurred in the control group and 12 in the experimental group. Of these, eight were classified as serious adverse events, which included left-hand paralysis, suspicious lung lesion suggestive of metastatic malignant tumor, arrhythmia, thoracic metastasis of a malignant tumor, pathological fracture of the distal femur, and two deaths. None of these serious adverse events were directly related to the study intervention. The most frequently reported adverse events (64%) were sensory disturbances in the region innervated by the mental nerve. Other non-serious adverse events included bronchospasm, oroantral communication, diarrhea, upper limb edema, hematoma in the antecubital fossa, urinary tract infection, lip swelling related to an allergic reaction, gastrointestinal discomfort, and transient palsy of the marginal mandibular branch of the facial nerve.

## Discussion

The results of our study suggest that the use of L-PRF as an adjunct to surgery does not significantly improve clinical healing rates when compared to surgery alone. At six months, the proportion of patients achieving complete resolution was 92% (11/12) in the experimental group and 83% (15/18) in the control group (RR 1.1, 95% CI 0.84– 1.43, *p*=0.51). Several studies have reported high resolution rates using L-PRF (10-18). A systematic review and meta-analysis on the use of L-PRF as an adjunct to surgery for the treatment of MRONJ, Muñoz Salgado *et al*. reported a weighted proportion of 94.3% complete resolution (95% CI: 91.2–97.4, *p*<0.001) (19). This review included ten studies: four cohort studies (14-18), two case series (18), one randomized clinical trial (10), and the remaining non-randomized clinical trials (10-12). The only randomized clinical trial compared surgical debridement combined with L-PRF and bone morphogenetic protein to surgical debridement with L-PRF alone (10). Complete resolution rates in patients who received L-PRF ranged from 69.2% to 100% in cohort studies and case series (14-17), and from 85% to 96.7% in clinical trials (10-12). Consistent with the literature, our study found high clinical resolution rates in the group that received surgery combined with L-PRF. However, no significant differences were observed when compared to the resolution rates in the surgery-only group.

Several authors have reported the negative impact of MRONJ on quality of life and oral health-related quality of life in oncologic and osteoporotic patients (20). In our study, we used the EQ-5D-3L questionnaire to assess baseline quality of life and the effect of the intervention on quality of life. Considering both groups together, the median baseline self-reported health status score was 60 (95% CI: 50–80) on a scale from 0 to 100. In line with the findings of Miskal *et al*. (21), the domains most affected in the baseline quality of life questionnaire in our study were anxiety/depression and pain/discomfort. No significant differences were observed between the experimental and control groups at six months postoperatively in any of the EQ-5D-3L domains. Other authors have reported that surgical treatment alone or in combination with adjunctive therapies improves quality of life in patients with MRONJ (20). Consistent with these findings, our study demonstrated an improvement in all domains when considering both groups together, particularly in the pain/discomfort and anxiety/depression domains.

Our study has some limitations. It was not possible to achieve the initially calculated sample size. While this reduces the study’s power (i.e., the ability to detect a true effect or difference between groups if one exists), the clinical resolution rates of MRONJ lesions in both groups were consistent with those reported in the literature, including primary clinical studies and systematic reviews with meta-analyses. Another limitation was the loss to follow-up at six months. The loss to follow-up rate was higher in the experimental group (36%) compared to the control group (18%), which introduces a significant risk of attrition bias. The higher loss to follow-up coincided with the COVID-19 pandemic period.

To the best of our knowledge our study is the first randomized controlled trial comparing surgery plus L-PRF with surgery alone for the treatment of MRONJ.

The results of our study suggest that the use of L-PRF as an adjunct to surgery does not provide a significant clinical benefit compared to surgery alone. No significant differences were observed between the experimental and control groups at six months postoperatively in any of the domains of the EQ-5D-3L questionnaire.

## Figures and Tables

**Table 1:** Baseline characteristics of study population and baseline quality of life (EQ-5D-3L).

	No. of patients (%)			p value
	Both groups (N=41)	Surgery alone (N=22)	Surgery + L-PRF (N=19)	
Age, median (IQR) [years]	73 (68, 77)	71 (64, 75)	74 (71, 78)	0.036
Female sex	37 (92%)	18 (86%)	19 (100%)	0.087
General risk factors				
Chemotherapy	18 (44%)	11 (50%)	7 (37%)	0.4
Age over 70 years	28 (68%)	12 (55%)	16 (84%)	0.042
Current smoker	9 (22%)	5 (23%)	4 (21%)	0.9
Former smoker	12 (29%)	8 (36%)	4 (21%)	0.28
Hyperthyroidism	5 (12%)	4 (18%)	1 (5%)	0.21
Type 2 diabetes	8 (20%)	6 (27%)	2 (11%)	0.18
Corticosteroid therapy in the last 6 months	9 (22%)	6 (27%)	3 (16%)	0.38
Local risk factors				
Periodontal disease	32 (78%)	16 (73%)	16 (84%)	0.38
Dental caries	30 (73%)	17 (77%)	13 (68%)	0.52
Poor oral hygiene	28 (68%)	16 (73%)	12 (63%)	0.51
Use of removable dentures	29 (71%)	14 (64%)	15 (79%)	0.28
Site				
Maxilla	7 (17%)	4 (18%)	3 (16%)	0.84
Mandible	34 (83%)	18 (82%)	16 (84%)	
Reason for antiresorptive therapy				
Osteoporosis	20 (49%)	11 (50%)	9 (47%)	0.25
Osteopenia	5 (12%)	1 (5%)	4 (21%)	
Bone metastasis	16 (39%)	10 (45%)	6 (32%)	
Antiresorptive medication				
Alendronate	6 (15%)	4 (18%)	2 (11%)	0.54
Ibandronate	17 (41%)	7 (32%)	10 (53%)	
Pamidronate	3 (7%)	1 (5%)	2 (11%)	
Zoledronic acid	10 (24%)	7 (32%)	3 (16%)	
Denosumab	5 (12%)	3 (14%)	2 (11%)	
Duration of antiresorptive treatment, median (IQR) [Months]	59 (24, 84)	57 (20, 73)	61 (25, 97)	0.29
Drug holiday	35 (85%)	21 (95%)	14 (74%)	0.049
Baseline characteristics of MRONJ lesions				
Pain, median (IQR)	50 (0-70)	50 (10-80)	30 (0-60)	0.35
Infection	37 (90%)	19 (86%)	18 (95%)	0.37
Bone exposure, median (IQR) [mm]	2 (0, 15)	0 (0, 10)	5 (0, 20)	0.12
Extraoral fistula	10 (24%)	5 (23%)	5 (26%)	0.79
AAOMS stage				
Stage 1	3 (7%)	2 (9%)	1 (5%)	0.9
Stage 2	17 (41%)	9 (41%)	8 (42%)	
Stage 3	21 (51%)	11 (50%)	10 (53%)	
Baseline quality of life (EQ-5D-3L)				
Mobility				
I have no problems walking	23 (56%)	11 (50%)	12 (63%)	0.4
I have some difficulty walking	18 (44%)	11 (50%)	7 (37%)	
Self-care				
I have no problems with self-care	34 (85%)	18 (86%)	16 (84%)	0.54
I have some difficulty washing or dressing myself	5 (12%)	2 (10%)	3 (16%)	
I am unable to wash or dress myself	1 (2%)	1 (5%)	0 (0%)	
Usual activities				
I have no problems with my usual activities	26 (63%)	11 (50%)	15 (79%)	0.11
I have some problems with my usual activities	13 (32%)	9 (41%)	4 (21%)	
I am unable to perform my usual activities	2 (5%)	2 (9%)	0 (0%)	
Pain/ discomfort				
I have no pain or discomfort	10 (24%)	4 (18%)	6 (32%)	0.29
I have moderate pain or discomfort	20 (49%)	10 (45%)	10 (53%)	
I have extreme pain or discomfort	11 (27%)	8 (36%)	3 (16%)	
Anxiety/ depression				
I am not anxious or depressed	7 (17%)	3 (14%)	4 (21%)	0.59
I am moderately anxious or depressed	20 (49%)	10 (45%)	10 (53%)	
I am extremely anxious or depressed	14 (34%)	9 (41%)	5 (26%)	
Current health status EQ-5D-3L, median (IQR)	60 (50, 80)	54.5 (35,70)	75 (50, 80)	0.032

L-PRF: Leukocyte and platelet rich fibrin. AAOMS stage: American Association of Oral and Maxillofacial Surgeons stage; MRONJ: Medicine related osteonecrosis of the jaws; IQR: Interquartile range. mm: millimeters. EQ-5D-3L: Euroquol 5D-3L quality of life questionnaire.

**Table 2:** Complete resolution at six-month follow-up.

	Both groups (N=30)	Surgery alone (N=18)	Surgery + L-PRF (N=12)	p value
Complete resolution, N (%)	26 (86%)	15 (83%)	11 (92%)	0.51

L-PRF: Leukocyte and platelet rich fibrin.

**Table 3:** Individual clinical domains at six-month follow-up.

Variable	Surgery alone (N=18)	Surgery + L-PRF (N=12)	p value
Pain (NPRS), median (IQR)	0 (0- 0)	0 (0- 0)	0.81
Bone exposure, median (IQR) [mm]	0 (0- 0)	0 (0- 0)	0.73
Extraoral fistula, N (%)	0 (0%)	1 (8%)	0.21
Infection, N (%)	2 (11%)	1 (8%)	0.80

L-PRF: Leukocyte and platelet-rich fibrin. NPRS: Numeric Pain Rating Scale (0- 100). IQR: Interquartile range. mm: millimeters.

**Table 4:** EQ-5D-3L quality of life assessment at six-month follow-up.

	No. of patients (%)			p value
	Both groups (N=41)	Surgery alone (N=22)	Surgery + L-PRF (N=19)	
Mobility				
I have no problems walking	20 (67%)	11 (61%)	9 (75%)	0.43
I have some difficulty walking	10 (33%)	7 (39%)	3 (25%)	
Self-care				
I have no problems with self-care	27 (90%)	16 (89%)	11 (92%)	0.69
I have some difficulty washing or dressing myself	2 (7%)	1 (6%)	1 (8%)	
I am unable to wash or dress myself	1 (3%)	1 (6%)	0 (0%)	
Usual activities				
I have no problems with my usual activities	22 (73%)	11 (61%)	11 (92%)	0.064
I have some problems with my usual activities	8 (27%)	7 (39%)	1 (8%)	
Pain/ discomfort				
I have no pain or discomfort	24 (80%)	16 (89%)	8 (67%)	0.082
I have moderate pain or discomfort	3 (10%)	0 (0%)	3 (25%)	
I have extreme pain or discomfort	3 (10%)	2 (11%)	1 (8%)	
Anxiety/ depression				
I am not anxious or depressed	15 (50%)	10 (56%)	5 (42%)	0.75
I am moderately anxious or depressed	13 (43%)	7 (39%)	6 (50%)	
I am extremely anxious or depressed	2 (7%)	1 (6%)	1 (8%)	
Current health status EQ-5D-3L, median (IQR)	80 (60- 90)	75 (60, 90)	80 (65, 90)	0.58

EQ-5D-3L: Euroquol 5D-3L quality of life questionnaire. IQR: interquartile range.

## Data Availability

The datasets used and/or analyzed during the current study are available from the corresponding author.
